# From Newsroom to Classroom

**DOI:** 10.1007/s12052-009-0144-1

**Published:** 2009-06-24

**Authors:** Anastasia Thanukos

**Affiliations:** University of California Museum of Paleontology, 1101 Valley Life Sciences Building, Berkeley, CA 94720-4780 USA

**Keywords:** Teaching, Curricula, Applications of evolution

Want a great example of evolution for your classroom? If we are guided by our textbooks, we should look to the radiation of Darwin’s finches (Grant and Grant 2008), the return of whales to the water (Thewissen 1998), the evolution of modern horses from their tiny ancestors (MacFadden 2005), or some similar well-established example for a case study. But are these sorts of illustrations the most compelling to students? Though such classic examples of evolution have more than earned their keep in the biology classroom, they are also removed from students’ everyday lives. *Hyracotherium*, for example, may fascinate some students but leave others wondering why they need to know this stuff. Just how relevant is a 50-million-year-old horse to a 14-year-old’s fast-paced media-driven world?

For this curriculum-themed issue, “Views from Understanding Evolution” departs from our usual format to introduce Evo in the News (Fig. 1)—a collaborative project of the UC Museum of Paleontology and the National Evolutionary Synthesis Center (NESCent). Evo in the News aims to help high school and college teachers bring current and relevant examples of evolution and evolutionary research into their classrooms to help teach basic concepts in evolutionary biology. Archives are freely available on the Understanding Evolution website (http://evolution.berkeley.edu/), and the latest updates can be received via a free subscription service (http://evolution.berkeley.edu/evolibrary/subscribe/email_signup.php).

**Fig. 1:**
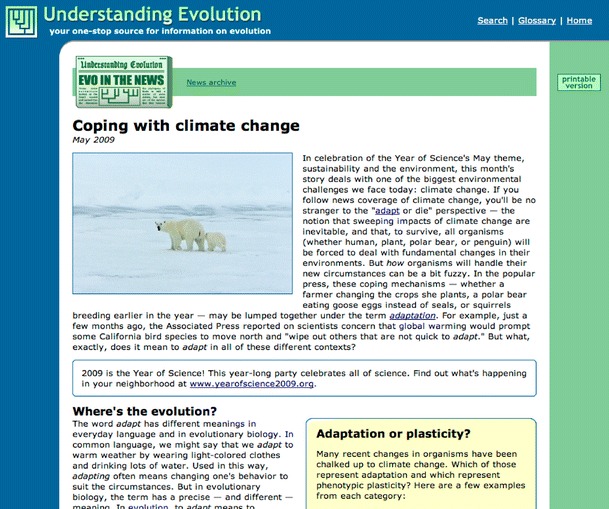
May 2009 edition of Evo in the News. This article explored the difference between phenotypic plasticity and evolutionary change in relation to popular reporting on climate change

## Current Events, not Ancient History

Educational research advocates engaging students by showing them how important concepts can be used to make sense of real problems and situations (National Research Council 1999). Using examples with direct links to students’ lives may raise their interest in the topic (Hillis 2007), increase their motivation to learn (National Research Council 2003), and encourage them to pursue science, technology, engineering, and math careers (STEM), as urgently recommended to bolster America’s scientific and technological infrastructure (Project Kaleidoscope 2006). Unfortunately, bringing current issues and events into the science classroom is often a challenge. Textbooks aim to keep up with advances in the field, but they are often thwarted by the rapid pace at which science moves. Scientific journals that describe cutting-edge, relevant evolutionary research come out daily—but it is difficult for most science teachers to translate these often complex and jargon-filled articles into something appropriate for the classroom. And, at the most basic level, the concerns and issues of interest to students and society are continually evolving. By the time that instructors have access to science teaching materials leveraging student interest in, for example, an emerging virus like swine flu or SARS, the threat and interest level are likely to have waned. How is a teacher to keep up?

The Evo in the News program circumvents these problems and lag times by providing a freely available suite of teaching resources (including lessons, readers, and video podcasts) on basic evolutionary concepts that capitalize on current events and come out on a monthly basis during the school year. Some Evo in the News briefs cover a hot topic in evolutionary research that has made it into the popular press in the preceding month (e.g., the discovery of *Tiktaalik* or new research on the origins of HIV; Daeschler et al. 2006; Worobey et al. 2008). In these cases, Evo in the News provides an explanation of the research in student-friendly terms, addresses the basic evolutionary concepts behind the research, and explains its relevance and importance. Of course, when some outlets in the popular media distort or misrepresent evolutionary research (e.g., when the discovery of a new hominid fossil is erroneously heralded as “challenging evolutionary theory”; Borenstein 2007; Spoor et al. 2007), Evo in the News explains the science behind the hype and why particular evolutionary issues are likely to be misinterpreted. Other news briefs address issues in the news that do not at first seem to have anything to do with evolution to reveal the “evolution behind the scenes”—which frequently gets short shrift in the popular press. For example, during National Breast Cancer Awareness Month, Evo in the News provided an evolutionary perspective on cancer; after major methicillin-resistant *Staphylococcus aureus* (MRSA) outbreaks, Evo in the News explained the evolution of this bacterial strain; and when six medical workers were sentenced to death in a Libyan trial, Evo in the News dealt with the evolutionary evidence that should have exonerated them. All concepts and news stories addressed are appropriate for high school, advanced placement biology, and/or college biology classes.

More than thirty-five Evo in the News stories are currently archived on the Understanding Evolution website (http://evolution.berkeley.edu/evolibrary/news/newsarchive_01). They address current events as wide-ranging as genetic engineering, conservation legislation, disease outbreak, DNA fingerprinting for criminal prosecution, and chemical engineering. Through the lens of these current events, the news briefs deal with fundamental evolutionary concepts, such as natural selection, mutation, genetic drift, speciation, evolutionary fitness, and phylogenetics. In the summer months, updates are added to these stories to keep pace with recent developments and maintain their relevance as an archived resource.

## Tools for Teaching

**Fig. 2:**
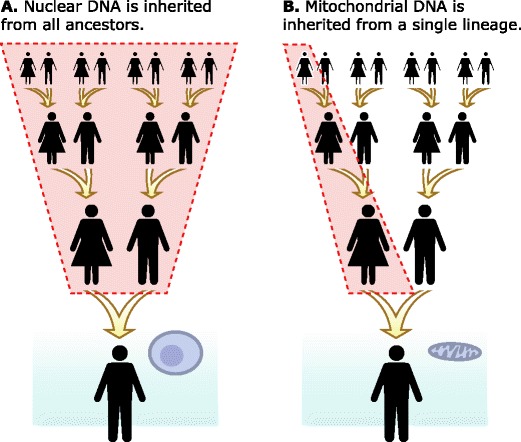
Explanatory graphic from the November 2007 edition of Evo in the News. This article explained the evolution behind genetic ancestry tests. **a** Ancestral sources of nuclear DNA. **b** Ancestral sources of mitochondrial DNA. Illustration reproduced with permission from the Understanding Evolution website

Each Evo in the News item comes with a set of freely available supplemental materials to help teachers integrate the item into their curricula. In addition to glossary definitions and explanatory graphics (Fig. 2), each news brief comes with:

- Links to freely available news and journal articles—students who may not be familiar with the current event that is the subject of a particular article can use popular press articles to get background information. Journal articles, when available, provide access to original scientific research.
- Links to Understanding Evolution resources—these readers and interactive features are written for students and provide additional background information on relevant evolutionary concepts.
- Discussion and extension questions—these questions can be used to stimulate class discussion on a news brief or can be assigned to students individually or in groups.
- Lessons and teaching resources—these freely available lessons have been vetted by a panel of teachers and can be used to relate the news item to other aspects of the curriculum or give students extra help learning particular key concepts.

**Fig. 3:**
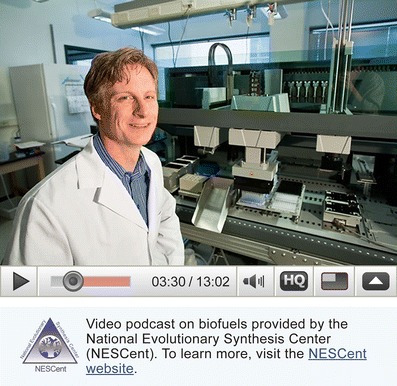
Podcast from the April 2009 edition of Evo in the News featuring Sydnor Withers of the Great Lakes Bioenergy Research Center

In addition, recent news briefs are supplemented by a short video podcast produced by NESCent. These podcasts vary in format slightly, but most are between five and fifteen minutes long and consist of a brief introduction to the topic, followed by an interview with a scientist whose research is closely related to the topic of the news brief. For example, the April 2009 story on the evolution behind biofuel production featured an interview with Sydnor Withers of the Great Lakes Bioenergy Research Center, who explained how his laboratory uses directed evolution to develop ways of building biofuels more efficiently (Fig. 3). These interviews provide students with a glimpse into the process of science and help give science a human face, while reinforcing central evolutionary concepts discussed in the news brief.

## Evo in the News in Your Classroom

The tools described above are designed to ease classroom implementation by providing teachers with a flexible suite of resources that they can deploy according to their needs and preferences. Some teachers may wish to use Evo in the News as it comes out every month, in order to bring current events into the classroom and incorporate evolution throughout the semester or school year. Others may choose to match selected news items to particular topics in their curricula. For example, a lesson about how genes code for proteins might be supplemented by the April 2007 news story on the evolution of lactose tolerance, which addresses the same topic; a lesson on the immune system could be supplemented by the March 2007 news story on why HIV presents such a challenge to vaccine developers; and a lesson on classification could be supplemented by the March 2008 story on the discovery of a new mammal species and how the classification of that group of organisms has changed over time as scientists have learned more about their evolutionary history. To locate Evo in the News items appropriate for a specific topic, visit the Understanding Evolution general lesson search engine (http://evolution.berkeley.edu/evosite/search/search.php), enter the topic of interest in the keyword search box, and select resources tagged “Evo in the News” (Table 1). To locate Evo in the News items that deal with particular evolutionary concepts, visit the concept search engine, find the grade level and concept you are interested in teaching, select “See Lessons,” and view resources tagged “Evo in the News” (Table 2).

**Table 1:** Evo in the News articles retrieved by general searches for the topics of speciation, behavior, DNA, and biodiversity

Search term	Articles	Publication date
Speciation	Evolving conservation strategies	June 2007
	Happy 200th, Darwin!	February 2009
	Sex, speciation, and fishy physics	Marcy 2009
Behavior	Cheating cheetahs prosper	July 2007
	Evolution’s dating and mating game	May 2008
	Quick bites and quirky adaptations	October 2006
	Quick evolution leads to quiet crickets	December 2006
DNA	Evolution at the scene of the crime	March 2006
	Genealogy enthusiasts mine DNA for clues to evolutionary history	November 2007
	Ghosts of epidemics past	October 2008
	Got lactase?	April 2007
	Seeing the tree for the twigs	May 2007
Biodiversity	Evolving conservation strategies	June 2007
	Hotspots for evolution	June 2006
	Tough conservation choices? Ask evolution	December 2008
	Where species come from	November 2006

**Table 2:** Evo in the News articles retrieved by sample 9–12 concept searches

Concept: articles	Publication date
Depending on environmental conditions, inherited characteristics may be advantageous, neutral, or detrimental:	
Another perspective on cancer	October 2007
Evolution from a virus’s view	December 2007
Ghosts of epidemics past	October 2008
Got lactase?	April 2007
Quick evolution leads to quiet crickets	December 2006
Sex, speciation, and fishy physics	Marcy 2009
Superbug, superfast evolution	April 2008
The amount of genetic variation within a population may affect the likelihood of survival of the population; the less the available diversity, the less likely the population will be able to survive environmental change:	
Cheating cheetahs prosper	July 2007
Evolution down under	September 2008
Warming to evolution	July 2006
Evolution may occur as a result of genetic drift:	
Evolution at the scene of the crime	March 2006
The similarity of DNA nucleotide sequences can be used to infer the degree of kinship between species:	
Evolutionary evidence takes the stand	January 2007
The new shrew that is not	March 2008

Evo in the News can be used in the classroom in many different ways—as take-home assignments, as the topic of small group discussions, as a departure point for an activity or class discussion, or as the starting point for individual or group research projects. This might be as straightforward as having students read a news brief, watch the podcast as a class, and turn in their answers to the discussion questions. Other teachers might prefer a more elaborate and open-ended implementation. For example, over the course of a few weeks, students and their teacher could explore several different news briefs. The teacher could then provide students with a popular press story with a hidden evolution connection and ask students to research and write a short explanation of this connection. Or students could be challenged to find their own news stories with an evolution connection and present them to the class. The possibilities are wide-ranging, and the supplementary teaching tools that come with each news brief are designed to support a variety of implementations.

## Future Directions

Evo in the News has responded and will continue to respond to teacher needs. In a formative evaluation of the program (Rockman et al. 2005), teachers reported that they were likely to use Evo in the News in their classrooms but that the utility of the briefs could be improved with study questions and additional support materials. In response, discussion questions and links to supporting lessons, activities, and readers were added. In a small-scale survey of the podcast component of Evo in the News, both teacher and students gave the podcast a positive rating but reported preferring shorter podcasts. In response, podcast length has been adjusted.

A more formal assessment of Evo in the News is currently underway in collaboration with the University of North Carolina, Chapel Hill, School of Education. It aims to assess the accessibility of the program, its effectiveness at increasing student understanding of evolutionary concepts, and its effectiveness at communicating the relevance of evolutionary theory. This evaluation will provide critical feedback for future improvements in the program. Information about participating in the evaluation can be found on the NESCent and Understanding Evolution websites. To keep up-to-date with Evo in the News as it evolves, subscribe to Understanding Evolution’s free e-letter.

Evolutionary concepts are central to basic science literacy (American Association for the Advancement of Science Project 2061 1993) and, increasingly, have applications in people’s everyday lives (e.g., in making consumer choices about genetically modified foods, purchasing an antibacterial soap, or voting on conservation issues) and in scientific advancement in other fields (e.g., medicine, chemical engineering, and even software design). By bringing Evo in the News into your classroom, you can provide your students with a ready answer to why they need to know all this evolution stuff anyway: because evolution is everywhere—and because it really matters in our everyday lives!

## Useful Links

- Education and outreach at The National Evolutionary Synthesis Center: http://www.nescent.org/eog/
- Evo in the News archive: http://evolution.berkeley.edu/evolibrary/news/newsarchive_01
- Evo in the News subscription service: http://evolution.berkeley.edu/evolibrary/subscribe/email_signup.php
- Keyword search for lessons and news items: http://evolution.berkeley.edu/evosite/search/search.php
- Concept search for lessons and news items: http://evolution.berkeley.edu/evosite/Lessons/IIConcepts.php
